# The impact of interventions on management of frailty in hospitalized frail older adults: a systematic review and meta-analysis

**DOI:** 10.1186/s12877-020-01935-8

**Published:** 2020-12-03

**Authors:** Zahra Rezaei-Shahsavarloo, Foroozan Atashzadeh-Shoorideh, Robbert J. J. Gobbens, Abbas Ebadi, Gholamreza Ghaedamini Harouni

**Affiliations:** 1 Student Research Committee, School of Nursing and Midwifery, Shahid Beheshti University of Medical Sciences, Tehran, Iran; 2 Department of Psychiatric Nursing and Management, School of Nursing and Midwifery, Shahid Beheshti University of Medical Sciences, Tehran, Iran; 3 Faculty of Health, Sports and Social Work, Inholland University of Applied Sciences, Amsterdam, The Netherlands; 4 Zonnehuisgroep Amstelland, Amstelveen, The Netherlands; 5 Department of Primary and Interdisciplinary Care, Faculty of Medicine and Health Sciences, University of Antwerp, Antwerp, Belgium; 6 Behavioral Sciences Research Center, Life style institute, Baqiyatallah University of Medical Sciences, Tehran, Iran; 7 Nursing Faculty, Baqiyatallah University of Medical Sciences, Tehran, Iran; 8 Social Welfare Management Research Center, University of Social Welfare and Rehabilitation Sciences, Tehran, Iran

**Keywords:** Frailty, Hospitalization, Frail elderly, Intervention, Systematic review; meta-analysis

## Abstract

**Background:**

One of the most challenging issues for the elderly population is the clinical state of frailty. Frailty is defined as a cumulative decline across psychological, physical, and social functioning. Hospitalization is one of the most stressful events for older people who are becoming frail. The aim of the present study was to determine the effectiveness of interventions focused on management of frailty in hospitalized frail older adults.

**Methods:**

A systematic review and meta-analysis of research was conducted using the Medline, Embase, Cochrane, ProQuest, CINAHL, SCOPUS and Web of Science electronic databases for papers published between 2000 and 2019. Randomized controlled studies were included that were aimed at the management of frailty in hospitalized older adults. The outcomes which were examined included frailty; physical, psychological, and social domains; length of stay in hospital; re-hospitalization; mortality; patient satisfaction; and the need for post discharge placement.

**Results:**

After screening 7976 records and 243 full-text articles, seven studies (3 interventions) were included, involving 1009 hospitalized older patients. The quality of these studies was fair to poor and the risk of publication bias in the studies was low. Meta-analysis of the studies showed statistically significant differences between the intervention and control groups for the management of frailty in hospitalized older adults (ES = 0.35; 95% CI: 0. 067–0.632; z = 2.43; *P* < 0.015). However, none of the included studies evaluated social status, only a few of the studies evaluated other secondary outcomes. The analysis also showed that a Comprehensive Geriatric Assessment unit intervention was effective in addressing physical and psychological frailty, re-hospitalization, mortality, and patient satisfaction.

**Conclusions:**

Interventions for hospitalized frail older adults are effective in management of frailty. Multidimensional interventions conducted by a multidisciplinary specialist team in geriatric settings are likely to be effective in the care of hospitalized frail elderly. Due to the low number of RCTs carried out in a hospital setting and the low quality of existing studies, there is a need for new RCTs to be carried out to generate a protocol appropriate for frail older people.

**Supplementary Information:**

The online version contains supplementary material available at 10.1186/s12877-020-01935-8.

## Background

Across the world, the elderly population is growing rapidly. It is expected that the elderly population will reach 2 billion people in 2050 [1] which raises serious concerns for the management and planning of health systems [2]. One of the most challenging issues for the elderly population is the clinical state of frailty [1]. Frailty is a new concept in medical sciences that is defined as “a clinically recognizable state of increased vulnerability that is the result of aging-related decline in function across multiple physiological organ systems such that the ability to cope with every day or acute stressors is compromised” [3]. There are two principal approaches to defining frailty, the frailty phenotype and the accumulation of deficits [4]. The phenotype approach of frailty uses the biological syndrome model, determining weight loss, exhaustion, weakness, low physical activity, and slowness. The accumulation of deficits recognizes that frailty results from an accumulation of abnormal features consisted of physical impairment, cognitive disorders, depressive symptoms, reduced functionality, multiple diseases, malnutrition, social isolation. In other words, physical features are a manifestation of frailty in phenotype approach, whereas causes of frailty indicate frailty in the accumulation of deficits [5, 6]. It is estimated that the incidence of frailty and pre-frailty in community-dwelling older adults are approximately 43 and 151 new cases per 1000 person-years, respectively [7].

Frailty refers to diminished physiological reserves to preserve homeostasis [3]. The frail older peoples are highly vulnerable adverse health outcomes when exposed to an internal or external stressor. One of the most stressful events for older people is hospitalization. It can be the cause of incidents which worsen the frailty of older people [8]. The prevalence of frailty in geriatric inpatients, depending on the evaluation tool used, ranges from 48.8 to 80% [9, 10]. Chen et al. (2019) demonstrated that the prevalence of hospitalized frail older adults, evaluated with Fried’s frailty phenotype, was 40% [11]. Frailty in hospitalized elderly predisposes them to falling [12], to delirium [13], to low quality of life [12], clinical deterioration [14], dependency [15], increase in length of hospital stay [16], poor recovery [17], ICU admission [18], institutional placement [14], rise in healthcare expenditure [19] and finally, frailty leads to the earlier death of patients [20].

Frailty is thought to be manageable in hospital with interventions such as physiotherapy [21], nutrition therapy [11], and comprehensive geriatric care (CGA) [22]. Several systematic reviews were conducted to determine the most effective interventions to reduce frailty in elderly people. Findings from previous systematic reviews showed a variety of interventions, including physical activity, cognitive training, nutrition therapy, CGA, group meetings, home visits, or a blend of these interventions [23–26]. Previous studies reported that physical activity is effective in improving frailty in older adults [24, 26]. However, these studies were delivered in the community, in primary care, and home care settings, rather than in hospital. No systematic review was found which focused on evidence with regard to interventions for hospitalized frail older adults, and so the present systematic review and meta-analysis was conducted. The aim was to determine the effectiveness of interventions focused on management of frailty in hospitalized older adults.

## Methods

### Study type

The present systematic review and meta-analysis was conducted based on the PRISMA guideline (Preferred Reporting Items for Systematic Reviews and Meta-Analyses( [27].

### Data sources and search strategy

Two independent researchers searched in the following electronic databases: Medline (via PubMed), Embase, Cochrane, ProQuest, CINAHL, SCOPUS and Web of Science for papers published between 1 January 2000 and 10 July 2019 with no language limitation. In addition, researchers carried out hand searches in the reference lists of obtained articles, including previous systematic reviews, to find further relevant studies. References to unpublished data were followed up to the main researcher who contacted the corresponding author or first author by email.

Frequently used phrases were identified using Medical Subject Headings (MeSH). The selected keywords were frail elders, functionally-impaired elderly, frailty syndrome, elderly, aged, 80 and over, prevention, intervention, effectiveness and outcome. These keywords were combined with appropriate Boolean operators, with each other or with other synonyms, and were searched in original peer-reviewed literature. Syntaxes were developed and completed in PubMed with a number needed to read (NNR) of 15 and were searched in other databases (Additional file 1).

### Study selection

Two researchers independently screened all potentially relevant studies by reading the titles and then the abstracts; disagreements were solved by discussion and using of the viewpoint of a third researcher.

### Inclusion and exclusion criteria

Relevance and appropriateness of the studies were assessed based on the inclusion and exclusion criteria. The inclusion criteria for this study were: clinical trial studies; regarding hospitalized patients “operationally defined as any patient admitted to hospital who remains overnight, or were initially expected to remain overnight”; age of samples ≥65 years; studies related to management, prevention, care, or cure of frailty; use of a validated operational definition of frailty “considering to phenotype of frailty and accumulation of deficits approaches”; and use of multidimensional specific frailty validated scale, measurement or index. The exclusion criteria were: pharmaceutical or pharmaceutical supplement interventions; frailty was not the outcome of the study; and intervention, program, model or protocol did not take place in a hospital setting.

### Data extraction

Two independent reviewers conducted data extraction. They extracted data with predetermined forms that were designed by the research team. The cases where there was no agreement were assessed by a third researcher. The data were extracted from all eligible studies, including: author names, publication year, geographical location, type of hospital, type of clinical setting, study design, recruitment duration, number of patients, mean and range age of samples, gender, inclusion and exclusion criteria, experimental conditions, control conditions, duration of intervention, operational definition of frailty, measured outcome, time-point of assessment, finding of primary and secondary outcomes, and evaluation of randomized programs or protocols.

### Assessment of risk of bias

The assessment of risk of bias was performed by two researchers independently using the Cochrane Risk of Bias Tool for clinical trial studies [28]. Disagreements were discussed with a third researcher and settled with a consensus decision. Researchers rated the quality of the included studies as “good”, “fair”, or “poor”. The selection studies were evaluated based on the criteria for clinical trials, accounting for study design and assessing potential for bias including “random sequence generation”, “allocation concealment”, “selective reporting”, “other sources of bias”, “blinding of participants and personnel”, “blinding of outcome assessment” and “incomplete outcome data” bias. Those articles with the highest risk for bias were defined as poor quality, and the studies with moderate and low risk of bias were considered as fair quality and good quality, respectively.

### Statistical analysis

A random-effect model was used to calculate changes in frailty from pre-to-post interventions. A forest plot was used for illustrating effect sizes and corresponding indexes. Egger’s test and funnel plots were used to assess publication bias. All meta-analytical methods were performed using STATA (Release 12. statistical software. College Station, Texas: STATA Corp LP).

### Outcome measures

The primary outcome of this systematic review was frailty. The secondary outcomes comprised physical, psychological, and social domains; length of stay in hospital; re-hospitalization; mortality**;** patient satisfaction; and the need for post discharge placement.

## Results

### Study selection

**Fig. 1:**
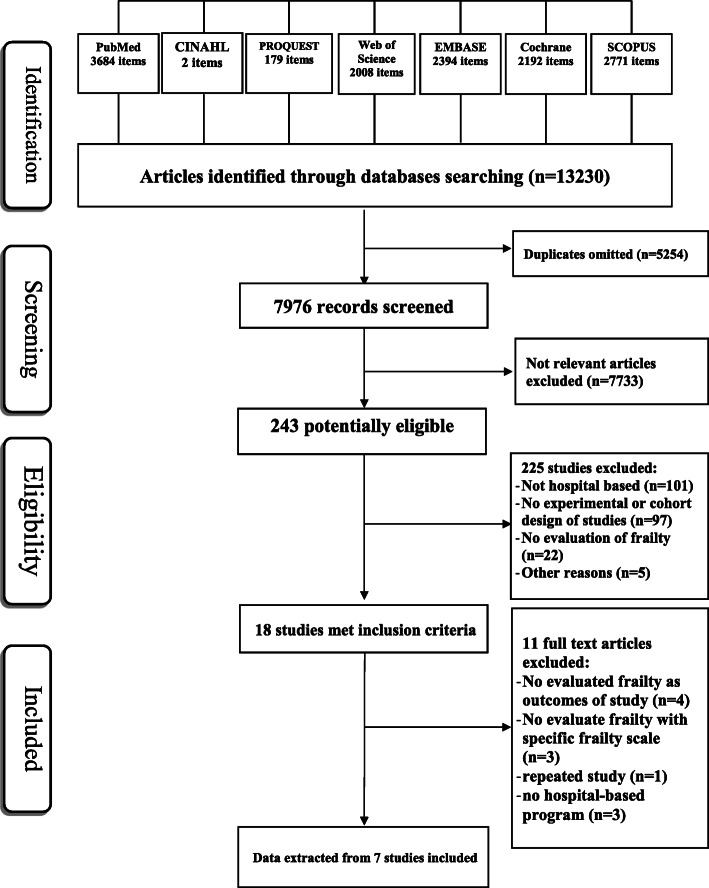
Flow diagram of search process

Figure 1 showed the search and selection of studies based on the PRISMA flowcharts. In databases search, 13,230 records were obtained. From 7976 non-duplicate records, the title and abstract of each study was screened of which 7733 were excluded and 243 with full text remained. From the 243 potentially eligible records, 18 studies met the inclusion criteria.

### Characteristics of included articles

Seven studies were included in the systematic review [11, 21, 22, 29–31]; four of these studies [22, 29, 31] reported outcomes of the same clinical trial project. One of four studies [22] reported about frailty and the three other studies [22, 29, 31] published secondary outcomes relevant for the present systematic review.

Table 1 showed that all of the included studies were randomized controlled trials. The sample size of the studies varied from 35 to 408 subjects. In total, these studies described 1009 hospitalized elderly patients; 527 patients belonged to the intervention group and 482 patients belonged to the control group; 50.3% of the participants were male. The mean age of the participants was between 72.8 (±5.6) to 85.7 (±5.4) years (Table 1).

**Table 1:** Description of included studies

Study	Geographicallocation; Type ofhospital, and ward	Study design	Samples	Inclusion criteria/exclusioncriteria	Recruitment duration
[29]	Västra GötalandRegion, Sweden; NU Hospital Group, acute medical careunit	clinical, prospective,controlled trial	Ni/Nc: 206/202 Mean ± SD:85.7 ± 5.4 gender, n(%):230 (56) Female	**Inclusion:** - Aged ≥75 years - need for in-hospital treatment - fulfilled criteria for frailty according FRESH screeninginstrument **Exclusion:** - patient clearly suited for care at an organ-specificmedical unit - informed consent could not be obtained	Mar. 2013 toJul. 2015
[32]	Västra GötalandRegion, Sweden; NU Hospital Group, acute medical careunit	clinical, prospective,controlled trial	Ni/Nc: 72/67 Mean ± SD, (intervention):85.6 ± 5.5 Mean ± SD, (control): 85.1 ± 5.6 gender, n(%): 47 (34) Male	**Inclusion:** - Aged ≥75 years - need for in-hospital treatment - fulfilled criteria for frailty according FRESH screeninginstrument **Exclusion:** - patient clearly suited for care at an organ-specificmedical unit	Mar. 2013 toJul. 2015
[31]	Västra GötalandRegion, Sweden; NU Hospital Group, acute medical careunit	clinical, prospective,controlled trial	Ni/Nc: 206/202 Mean ± SD: 85.7 ± 5.4 gender, n(%): 230 (56) Female	**Inclusion:** - Aged ≥75 years - need of in-hospital treatment - fulfilled the criteria for frailty according FRESHscreening instrument **Exclusion:** - Declined participation in the study - patient clearly suited for care at an organ-specificmedical unit - informed consent could not be obtained - from patients previously defined MÄVA patients - cognitively impaired patients	Mar. 2013 to Jul. 2015
[22]	Västra GötalandRegion, Sweden; NU Hospital Group, acute medical careunit	clinical, prospective,controlled trial	Ni/Nc: 206/202 Mean ± SD, (intervention):85.6 ± 5.5 Mean ± SD, (control): 85.1 ± 5.6 gender, n(%): 230 (56) Female	**Inclusion:** - Aged ≥75 years - need of in-hospital treatment - fulfilled the criteria for frailty according FRESHscreening instrument **Exclusion:** - patient clearly suited for care at an organ-specificmedical unit - from patients previously defined MÄVA patients	Mar. 2013 to Jul. 2015
[21]	Western part,Germany; Generalacademic teachinghospital, Acute medicalgeriatric ward	prospective, parallelgroup, randomizedcontrolled pilot andfeasibility trial	Ni/Nc: 17/18 Mean ± SD: 80.9 ± 7.7 gender, n(%):74% female	**Inclusion:** - a minimum age of 65 years - planned acute-geriatric stay of - at least two weeks in the study hospital - care plan according to “early rehabilitation ingeriatric medicine” (GFK) procedures - walking ability (with or without walking aid;independent or with stand-by assistance), indicatedby a Functional Ambulation Categories (FAC)score ≥ 3 - limited mobility, indicated by a timed up and gotest (TUG) score of > 9 s **Exclusion:** - significant cognitive impairment - severe hearing impairment - severe visual impairment - German language barrier - acute psychiatric condition (e.g.delirium) - initiated palliative care - any medical restriction for - physiotherapeutic interventions(e.g. physical training) - lack of understanding of simpleorders - first phase: baseline-assessmenthad not been completed withinthe first 5 days after hospitaladmission, second phase: baseline-assessmenthas not been completed within thefirst five days after initial physician’sprescription for physiotherapy	First phase: Oct. 2016 to Dec. 2016 sphase: Oct. 2017 to Dec. 2017
[11]	Taipei,Taiwan; university-affiliatedmedical center,GastrointestinalSurgery	Cluster randomizedtrial	Ni/Nc: 197/180 Mean ± SD: 74.5 ± 5.8 gender, n(%):214 (56.8) Male	**Inclusion:** - Age ≥ 65 years - admitted in 36-bed gastrointestinal ward of urban medical center - scheduled for elective abdominalsurgery - expected length of stay > 6 days **Exclusion:** - profound dementia - refused participation (patient,family, or physician refusal), - 42 were not enrolled because ofcritical/terminal illness, - respiratory isolation - Severe hearing or visual impairmentthat precluded communication.	Aug. 2009 to Oct. 2012
[30]	Taiwan; Gastrointestinal ward	Before and afterintervention study	Ni/Nc: 107/82 Mean ± SD, (intervention): 73.3 ± 6.2 Mean ± SD, (control): 72.8 ± 5.6 gender, n(%): 82 (43) Female	**Inclusion:** - Age ≥ 65 years - admitted in 36-bed gastrointestinal ward of urbanmedical center - scheduled for elective abdominal surgery - expected length of stay > 6 days - completed discharge and 3-month evaluations **Exclusion:** - present in the hospital at the time of the analysis - some form of data was missing - discharged to temporary accommodation ortransferred to another hospital	control group: Aug. 2007 to Apr. 2008 Intervention group: May 2008 to Apr. 2009

*NU NÄL-Uddevalla, *Ni/Nc* Number of intervention group/number of control group

The seven studies were carried out in hospitalized frail elderly in Sweden (*n* = 4) [22, 29, 31], Germany (*n* = 1) [21] and Taiwan (*n* = 2) [11, 30]. The studies were focused on hospitalized frail elderly in an acute medical ward (*n* = 4) [22, 29, 31], gastrointestinal surgery ward (*n* = 2) [11, 30], and acute medical geriatric ward (*n* = 1) [21]. Also, three studies had a follow-up of 3 months [22, 29], two studies had a follow-up at discharge [11, 30] and one study had a follow-up of 14 days to 3 weeks after baseline assessment [21] (Tables 1 and 2).

**Table 2:** Operational definition of frailty, measured outcomes and time point of assessment

study	Operational definition of frailty	measured outcome	Time point of assessment
[29]		**Physical fitness** - Handgrip Strength: hydraulic hand dynamometer - Functional mobility: TUG - submaximal aerobic capacity: 6-MWT **Length of stay**	**Baseline** - before discharge from index hospital stay **Follow up** - 3-month follow-up visit
[32]		**Satisfaction**	Filled in shortly after discharge of hospital
[31]		**Health related quality of life: (HUI-3)** - Hearing - Speech - Ambulation - Dexterity - Emotion - Cognition - Pain **EuroQoL-visual analog scale** **Re-hospitalization** **Mortality**	**Baseline** - before discharge from hospital **Follow up** - 1 month follow up of re-hospitalization - 3-month follow-up visit
[22]	Two or more of the following criteria: tiredness, falls, endurance, needing support while shopping and visits to the emergency department	**Frailty** - FRESH screening tool **Decline in functional activity** - ADL Staircase: Personal ADL Instrumental ADL **Increased use of municipal services**	**Baseline** - Index hospitalization **Follow up** - 3 months after discharge
[21]	Frailty Index was measured of frailty according to the model of deficit accumulation, 40 item Frailty Index was calculated based on proposed variables by Searle et al. the score of Frailty Index is the ratio of health deficits present to the total number of health-related variables. Peak flow, shoulder strength, grip strength and gait speed were rated based on actual physical performance. All other items were patient reported.	**Frailty:** - Frailty Index **Mobility:** - DEMMI - Gait speed - HABAM - TUG **Walking ability:** - Functional Ambulation Categories **Physical endurance:** - 6-MWT **Falls efficacy**: - Falls efficacy scale **Length of stay** **Adherence rate**	**Baseline** - First phase: 5 days after hospital admission - Second phase: 5 days after initial prescription for usual care **Follow up** - minimum of 14 days after hospital admission - maximum three weeks after baseline assessment
[11]	Frailty by meeting 4 out of 5 Fried’s criteria: - Unintended weight loss of more than 5% from the previous time point - Weakness (grip strength) - Self-report exhaustion - Low activity by esds - Slowness by ESDS	**Frailty:** - Fried’s criteria **Physical status:** - Body Weight	**Baseline:** - At admission **At discharge**
[30]	Frailty by meeting 4 out of 5 Fried’s criteria: - weight loss > 5% compared to previous time point - weakness by hand grip strength - self-report exhaustion - low activity level by ESDS - Slow walking speed by ESDS	**Frailty:** - Fried’s criteria **Length of stay**	**Admission** **Before discharge** **3 months after discharge**

*TUG* Timed up-and-go test, *6-MWT* 6-Mined Walked Test, *HUI-3* Health Utilities Index-3, *EQ-VAS* EuroQol-visual analog scale, *ADLs* Activity of Daily Living Staircase, *DEMMI* De Morton Mobility Index, *HABAM* Hierarchical assessment of balance, *ESDS* Enforce Social Dependency Scale

### Risk of bias assessment

None of the included studies fulfilled all of the quality criteria. All studies were poor in “Blinding of participants and personnel”. Moreover, the studies by Braun et al. (2019) Chen et al. (2018) and Chen et al. (2014) scored poorly on the criteria “other sources of bias” and “incomplete outcome data”. Only the quality of the study by Braun et al. (2019) was categorized as fair [21] and all other studies demonstrated poor quality [11, 22, 29–31] (Additional file 2).

### Dimensions of interventions in included studies

In seven clinical trial studies, three randomized intervention programs or protocols were conducted for the management of hospitalized frail elderly. Four studies implemented a CGA program for caring for hospitalized frail older adults. The intervention group received structured, systematic interdisciplinary CGA-based care at an acute elderly care unit [22, 29, 31]. Braun et al. (2019) implemented augmented a prescribed exercise program (APEP) in the intervention group. The core of APEP was additional individual physiotherapy without pre-defined protocol or set of exercises [21]. Chen et al. (2014) and Chen et al. (2018) conducted modified Hospital Elder Life Program (mHELP). The mHELP program had early mobilization, oral and nutritional assistance, and orientation communication [11, 30] (Additional file 3).

### Study outcomes

#### Frailty

From 7 included studies [11, 21, 22, 29–31], 4 studies reported findings from the same clinical trial [22, 29, 31]. Of these four studies, only the study by Ekerstad et al. (2017) [22] used frailty as an outcome. Furthermore, from the 7 included studies in the present systematic review, three studies that had not evaluated frailty were excluded, and four studies that reported frailty findings were imported into the meta-analysis [11, 21, 22, 30]. Ekerstad et al. (2017) assessed the degree of frailty with the FRESH screening tool [22]. Braun et al. [21] and Chen et al. [11, 30] measured frailty with the Frailty Index and the Fried criteria, respectively. The incidence of frailty and persistent frailty were evaluated by Chen et al. [11]. Also, transitions between frailty states and the rate of frailty were measured by Chen et al. [30].

**Fig. 2:**
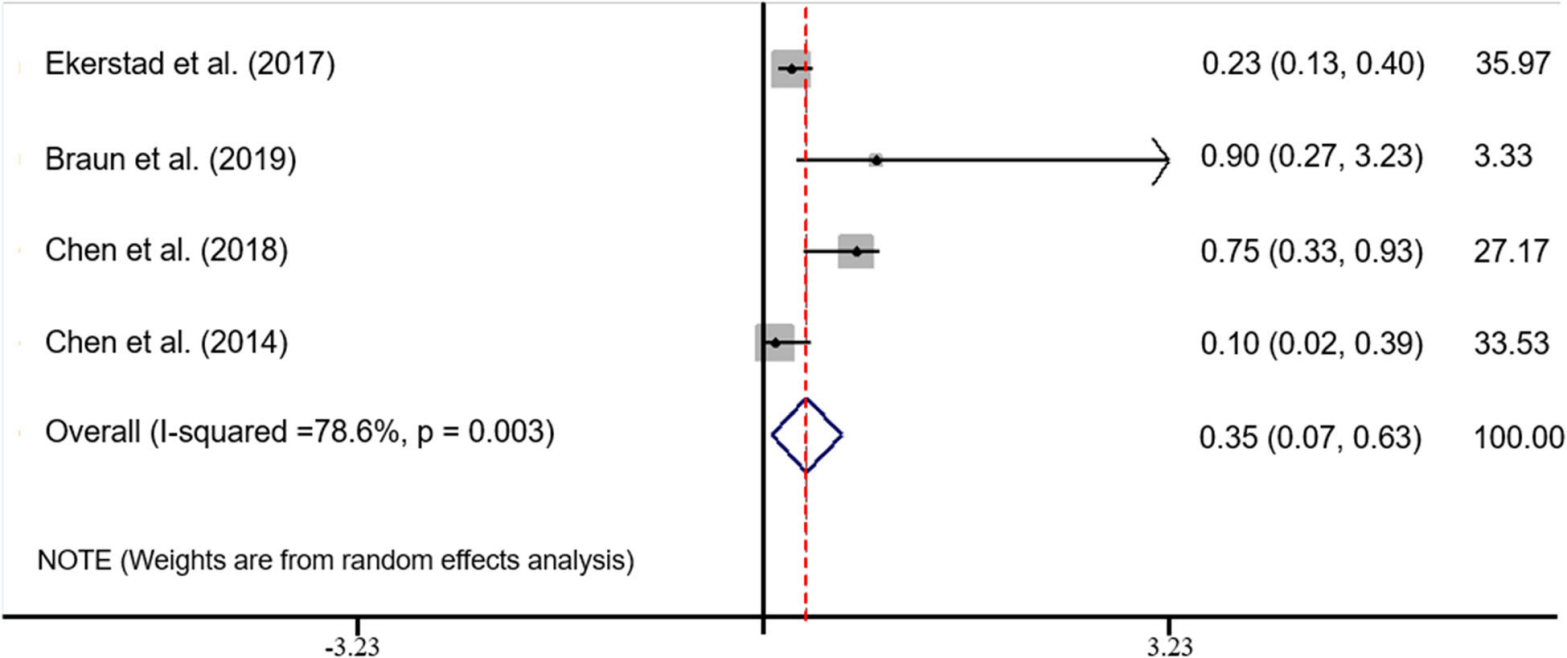
Forest plot for meta-analysis of all studies

Meta-analysis of the studies showed statistically significant differences between the intervention and control groups for management of frailty in hospitalized older adults (ES = 0.35; 95% CI: 0. 067–0.632; z = 2.43; *P* < 0.015). The I-squared was 78.6%, indicating a high degree of heterogeneity. According to the low number of studies, subgroup analysis wasn’t possible due to heterogeneity of the studies (Table 3 and Fig. 2).

**Table 3:** Meta-analysis results for all studies

Study	ES	95% CI		% Weight
		LB	UB	
[22]	0.229	0.131	0.400	35.97
[21]	0.900	0.270	3.230	3.33
[11]	0.750	0.330	0.930	27.17
[30]	0.100	0.020	0.390	33.53
D + L pooled ES	0.350	0.067	0.632	100

#### Physical domain

Two studies showed that CGA was effective in terms of Handgrip Strength (HS) (*p* < 0.001), 6-Minute Walk Test (6-MWT) (*p* < 0.001), Timed Up-and-Go test (TUG) (*p* = 0.042) decline in Activity Daily Living (ADL) Staircase [OR = 0.093; 95% CI (0.052–0.16)] and decline in ADL stratum (*p* = 0.0001) [22, 29]. One study reported that the mHLEP intervention group lost less weight (*p* = 0.002) compared with the control group [11]. However, Braun et al. showed that APEP improved the mean score on De Morton Mobility Index (DEMMI), TUG and 6-MWT, but these effects were not statistically significant, *p* = 0.26, *p* = 0.21, and *p* = 0.11, respectively [21] (Table 4).

**Table 4:** Primary and secondary outcomes of studies

study	Intervention /control condition	Primary Outcome-Frailty	Secondary outcomes	Significance
[29]	CGA unit (*N* = 206)		**At baseline**: - Physical fitness, mean ± SD: HS: 18.8 ± 7.2; 6-MWT: 146 ± 103.4; TUG: 30 ± 23.2 - Number of Hospital days: 11.2 **At follow up vs baseline:** - Physical fitness, mean (95% CI) ^a^: HS: + 1.64 (0.93–2.36); 6-MWT: + 21.4 (5.8–37); TUG: + 6.75 (4.03–9.45) - Decline in physical fitness, n(%), [OR (95% CI)]: HS: 23 (17.2), [3.2 (1.7–6.1)] ^a^; 6-MWT: 9 (10.8), [7.0 (2.8–17.7)] ^a^; TUG: 18 (17.1), [2.8 (1.3–5.9)] ^a^ Number of hospital days per patient, mean: 16.2	**Between group changes:** - change in physical fitness ^a^ HS *p* < 0.001 6-MWT *p* < 0.001 TUG *p* = 0.042 - Number of hospital days: *p* = 0.002
	Conventional acute care (*N* = 202)		**At baseline,** mean ± SD: - Physical fitness: HS: 18 ± 7.9; 6-MWT: 160 ± 100; TUG: 37.4 ± 28.6 - Number of Hospital days: 9.2 **At follow up vs baseline:** - Physical fitness, mean (95% CI) ^a^: HS: − 0.9 (− 1.7 to − 0.1); 6-MWT:: --60.7 (− 80.6 to − 40.9); TUG: + 2.19 (− 1.15 to 5.45) - Decline in physical fitness, n(%): HS: 46 (42.6); 6-MWT: 26 (50); TUG: 26 (37.1) Number of Hospital days, mean: 16.9	
[32]	CGA unit (*N* = 72)		**Follow up**, n (%): - Getting help from doctors with medical problems: Great help, fairy great help: 62 (86.1); Little and very little help: 10 (13.9) - Getting nursing from ward staff that you needed: Yes always, yes often: 66 (98.5); No not often, no seldom: 1 (1.5) - Satisfied with received information: Very satisfied, fairy satisfied: 64 (90.1); Fairy unsatisfied: 7 (9.1) Satisfied with planning before discharge**:** Very satisfied, fairy satisfied: 64 (89); Fairy unsatisfied: 8 (11)	**Between group changes:** - Getting nursing that you needed *p* = 0.003 - Satisfied with received information *p* = 0.016 Satisfied with planning before discharge *p* = 0.023
	Conventional acute care (*N* = 76)		**Follow up**, n (%): - Getting help from doctors with medical problems: Great help, fairy great help: 50 (75.8); Little and very little help: 16 (24.2) - Getting nursing from ward staff that you needed: Yes always, yes often: 55 (83.3); No not often, no seldom: 11 (16.7) - Satisfied with received information: Very satisfied, fairy satisfied: 50 (74.6); Fairy unsatisfied: 17 (25.4) Satisfies with planning before discharge**:** Very satisfied, fairy satisfied: 48 (74); Fairy unsatisfied: 17 (26)	
[31]	CGA unit (*N* = 206)		**At baseline** - HUI-3, mean: Vision: 0.886; Hearing: 0.815; Speech: 0.999; Ambulation: 0.540; Dexterity: 0.871; Emotion: 0.823; Cognition: 0.896; Pain: 0.621 - EQ-VAS score, mean: 51.1 - Mortality, n (%): 8 (4) **At follow-up (1 month**) - Rehospitalization, n (%): 40 (19) **At follow-up (3 months)** - HUI-3, mean: Vision: 0.873; Hearing: 0.818; Speech: 0.995; Ambulation: 0.584; Dexterity: 0.856; Emotion: 0.896; Cognition: 0.933; Pain: 0.766 - Decline in HUI, OR (CI 95%) ^a^: vision: 0.33 (0.14–0.79); ambulation: 0.19 (0.1–0.37); dexterity: 0.38 (0.19–0.75); emotion: 0.43 (0.22–0.84); cognition 0.076 (0.033–0.18); pain: 0.28 (0.15–0.50); hearing: 0.50 (0.22–1.1); speech: 0.45 (0.11–1.9) - EQ-VAS score, mean: 56.8 - Mortality, n (%), [HR (CI 95%)] ^a^: 27 (13), [0.55 (0.32–0.96)] Rehospitalization, n (%): 73 (37)	**Between group changes in follow-up:** - HUI-3: Ambulation *p* = 0.001; cognition *p* < 0.001; pain *p* < 0.001 - Decline in HRQoL: vision *p* = 0.013; ambulation *p* < 0.001, dexterity *p* = 0.007, emotions *p* = 0.014, cognition *p* < 0.001, and pain *p* < 0.001 - Rehospitalizations: *P* = 0.048 EQ-VAS score: *p* = 0.003
	Conventional acute care (*N* = 202)		**At baseline** - HUI-3, mean: Vision: 0.884; Hearing: 0.881; Speech: 0.975; Ambulation: 0.569; Dexterity: 0.882; Emotion: 0.865; Cognition: 0.877; Pain: 0.631 - EQ-VAS score, mean: 48.9 - Mortality, n (%): 10 (5) **At follow-up (1 month**) - Rehospitalization, n (%): 56 (28) **At follow-up (3 months)** - HUI-3, mean: Vision: 0.862; Hearing: 0.817; Speech: 0.985; Ambulation: 0.458; Dexterity: 0.804; Emotion: 0.896; Cognition: 0.834; Pain: 0.594 - EQ-VAS score, mean: 51.2 Rehospitalization, n (%): 88 (46)	
[22]	CGA unit (*N* = 206)	**At follow up vs baseline**: Increase in degree of frailty, assessed with FRESH screening tool, n (%), [OR (95% CI)]: 24 (13.6), [0.229 (0.131–0.400)]	**At follow up vs baseline** - Average change of ADL Staircase, mean ± SD: 0.2 ± 1.1 to up - Decline in ADLs staircase, n (%), [OR (95% CI)]: 24 (14.1), [0.093 (0.052–0.16)] ^a^ - Decline in ADL stratum, n(%): 11 (6.3) Increase in use of municipal services, n(%), [OR (95% CI)]: 36 (20), [0.682 (0.395–1.178)] ^a^	**Between group changes:** - Increase in degree of frailty *p* < 0.0001 - Decline in ADLs *p* < 0.0001 Decline in ADL stratum: *p* = 0.0001
	Conventional acute care (*N* = 202)	**At follow up vs baseline**: Increase in degree of frailty, assessed with FRESH screening tool, n (%): 66 (41)	**At follow up vs baseline**: - Average change of ADL Staircase, mean ± SD:1.1 ± 1.6 to down - Decline in ADL staircase, n(%): 98 (63.6) - Decline in ADL stratum, n(%): 33 (20.2) Increase in use of municipal services, n(%): 44 (26.2)	
[21]	APEP group (*n* = 17)	**Baseline:** - Frailty index, mean ± SD: 0.46 ± 0.20 **Follow up**, mean ± SD, [mean (95% CI)]: Frailty index: 0.40 ± 0.19, [0.01 (−0.02 to 0.05)] ^a^	**Baseline:** - Mobility, mean ± SD: DEMMI: 49.4 ± 16.0; HABAM: 19.1 ± 4.7; TUG: 28.6 ± 13.2; Gait Speed: 0.53 ± 0.17 - 6-MWT, mean ± SD: 154.5 ± 59.6 - FES-1, Median (IQR): 31 (22–57) - FAC, Median (IQR): 4 (3–4) **Follow up** Mobility, mean ± SD, [mean (95% CI)]: DEMMI: 57.2 ± 17, [4.1 (0.4 to 7.8)] ^a^; HABAM: 20.3 ± 4.9, [0 (− 0.9 to 0.9)] ^a^; TUG: 22.8 ± 12.2, [2.5 (0.4 to 4.6)]^a^; Gait Speed: 0.65 ± 0.20, [0.07 (0.01 to 0.13)]^a^ 6-MWT, mean ± SD, [mean (95% CI)]: 194.9 ± 85.8, [34.7 (13.7 to 55.7)] ^a^ FES-1, median (IQR), [mean (95% CI)]: 30 (22–52), [24 (−5.5 to 10.3)]^a^ FAC, median (IQR), [mean (95% CI)]: 4 (4–4), [0 (− 0.4 to 0.4)] ^a^ Length of stay, mean ± SD: 18.4 ± 2.3 Adherence rate, mean ± SD: 78 ± 26%	
	Usual care (*n* = 18)	**Baseline:** - Frailty index, mean ± SD: 0.46 ± 0.14 **Follow up:** Frailty index, mean ± SD: 0.41 ± 0.15	**Baseline:** - Mobility, mean ± SD: DEMMI: 52.9 ± 11.1; HABAM: 19.9 ± 4.4; TUG: 24.9 ± 11.1; Gait Speed: 0.60 ± 0.19 - 6-MWT, mean ± SD: 167.7 ± 79.4 - FES-1, Median (IQR): 31 (26–45) - FAC, Median (IQR): 4 (3–4) **Follow up,** mean (SD): - Mobility, mean ± SD: DEMMI: 55.7 ± 11.3; HABAM: 20.9 ± 4.0; TUG: 22.4 ± 9.5; Gait Speed: 0.64 ± 0.28 - 6-MWT, mean ± SD: 170.8 ± 79.9 - FES-1, Median (IQR): 31 (25–46) - FAC, Median (IQR): 4 (3–4) Length of stay, mean ± SD: 17.8 ± 4.2	
[11]	mHELP group (*n* = 197)	**At follow up**, n (%), [RR (95% CI)] - Incident frailty, assessed with Fried’s criteria, during stays in hospital: 20 (12), [0.55 (0.33–0.93)] Persistent frailty: 6 (50), [0.54 (0.30–0.97)]	**Follow up** Changes on body weight, mean ± SD: − 2.1 ± 5.5	**Between group changes:** - Incident frailty during stays *p* = 0.02 - Persistent frailty *p* = 0.03 Changes on body weight: *p* = 0.002
	Usual care (*n* = 180)	**At follow up**, n (%): - Incident frailty, assessed with Fried’s criteria, during stays in hospital: 30 (21.7) Persistent frailty: 13 (92.9)	**Follow up** Changes on body weight, mean ± SD: − 4.0 ± 3.4	
[30]	mHELP group, *n* = 107	**At discharge** - Transitions between Fried’s frailty states of pre-frail: advanced to frail: 18%; pre-frail: 64%; non frail: 18% - Rate of frailty using Fried’s criteria, n(%), [OR (95% CI)] ^b^: 10.52 (19.2), [0.1 (0.02–0.39)] **Follow up** - Rate of frailty according to Fried’s criteria, n(%), [OR (95% CI)] ^b^: 9.52 (17.3), [0.73 (0.21–2.56)] Improved to not frail according to Fried’s criteria: 21%	**Follow up:** Length of hospital stay (days), mean ± SD: 20.5 ± 18.2	**Between group changes:** - Difference in Transitions between frailty states, *p* < 0.001 Frailty rate at discharge; *p* = 0.001
	Usual care, *n* = 82	**At discharge** - Transitions between Fried’s frailty states of pre-frail: advanced to frail: 68%; remaining in a pre-frail: 32% - Rate of frailty using Fried’s criteria, n(%): 34.52 (65.4) ^b^ **Follow up** - Rate of frailty according to Fried’s criteria, n(%): 12.52 (23.1^)b^ Improved to not frail according to Fried’s criteria: 9%	**Follow up:** Length of hospital stay (days), mean ± SD: 17.3 ± 11.0	

^a^adjusted analysis, ^b^adjusted analysis and matched pairs, *HS* Handgrip Strength, *6-MWT* 6-Mined Walked Test, *TUG* Timed up-and-go test, *HUI-3* Health Utilities Index-3, *EQ-VAS* EuroQol-visual analog scale, *DEMMI* De Morton Mobility Index, *HABAM* Hierarchical assessment of balance, *FES- І* Falls efficacy, *FAC* Functional Ambulation Categories

#### Psychological and social domains

None of studies evaluated the social domain of patients and only one of them reported the psychological domain by health-related quality of life scale (HRQoL) [22]. Ekerstad et al. (2017) found that the control group experienced a significantly higher decline in HRQoL compared to the intervention group with regard to vision, ambulation, dexterity, emotions, cognition and pain dimensions (*p* < 0.01). In addition, this study reported a higher EuroQol-visual analog scale (EQ-VAS) score in intervention group than in the control group (*p* = 0.003) [22] (Table 4).

### Hospitalization status

#### Length of stay

Three studies evaluated length of stay in the hospital. Two studies showed no significant differences between the intervention and control groups [21, 30]. The single study that used CGA demonstrated that length of stay was significantly higher in the intervention group compared with the control group [29] (Table 4).

#### Re-hospitalization

One study reported the re-hospitalization rate. This study showed a lower prevalence of re-hospitalization rate in the CGA unit than in the conventional care unit 1 month after discharge (*p* = 0.048), but after 3 months of discharge there was not a significant difference in re-hospitalization between the two units [22] (Table 4).

#### Mortality

Only the study by Ekerstad et al. (2017) was focused on the effect of the interventions on the mortality rate of the participants. The mortality rate was lower in the CGA unit intervention group than in the conventional care unit group [HR = 0.55; 95% CI (0.32–0.96)] [22] (Table 4).

### Satisfaction with program

The study by Ekerstad et al. (2018) showed that the group receiving the intervention (CGA) was more satisfied with the program than the control group. The intervention group scored significantly higher on satisfaction with received information (*p* = 0.016) and satisfaction with planning before discharge (*p* = 0.023) [31] (Table 4).

### Post discharge placement

One study reported that the control group used more supplementary services compared with the intervention group patients [OR = 0.682; 95% CI (0.395–1.178)] [22] (Table 4).

### Publication bias assessment

**Fig. 3:**
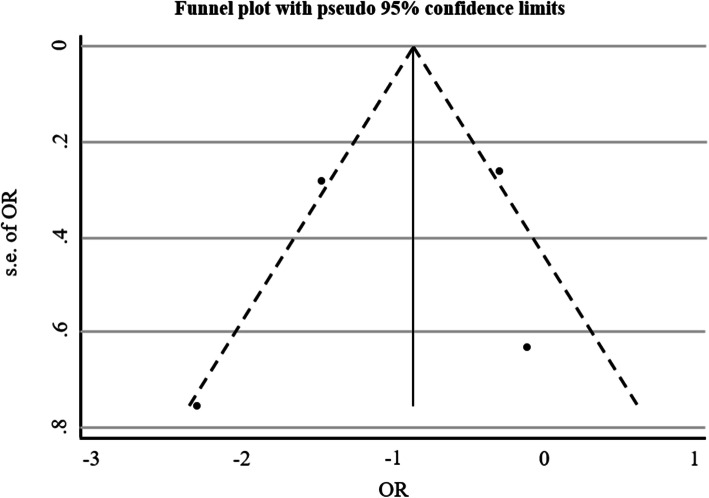
Funnel plot for all studies publication bias

Egger regression analysis were applied to analyze publication bias. The results showed that the risk of bias was low (*p* = 0.063) (Table 5 and Fig. 3).

**Table 5:** Egger Results for Publication Bias

Std_Eff	Coef.	Se	t	***P*** > t	95% CI	
					LB	UB
**slope**	.0542103	.0229963	2.36	0.142	−.0447349	.1531554
**bias**	2.216192	.5841663	3.79	0.063	−.2972728	4.729657

## Conclusions

This systematic review and meta-analysis was conducted to evaluate the effectiveness of interventions for the care and management of frailty in hospitalized older adults. Moreover, the present study aimed to determine the effectiveness of interventions on physical, psychological, social domains, and hospitalization status. Four of these studies were conducted in Sweden and reported outcomes of the same clinical trial [22, 29, 31]. The other studies were carried out in Germany [21] and Taiwan [11, 30].

Our study showed that interventions are effective in the management of frailty in hospitalized older adults. Other systematic review studies published paradoxical findings for example, Arantes et al. (2009) concluded that the effectiveness of physical therapy on frailty among community-dwelling elderly was low [32]. However, Negm et al. (2019) and Apóstolo et al. (2019) showed that interventions based on physical activity and a combination of physical activity and nutritional supplementation was possibly the most effective measure in decreasing frailty [23, 26]. It appears that different contexts and diversity of interventions are probably the reason of paradoxical findings. Also, it seems that multidimensional interventions covering physical, psychological and social functioning of hospitalized frail older patients are more effective than one dimension interventions [33]. This is supported by a study using the APEP intervention [21], which was targeted on individual physiotherapy and didn’t consider the social and psychological aspects of frail older adults; APEP was less effective on the degree of frailty. However, further clinical trials studies with long-term follow up are needed to confirm this hypothesis.

Our study showed that CGA was effective on frailty in the physical domain. Moreover, the mHELP intervention was effective on prevention of weight loss in the intervention group [11, 22, 29]. Also, Braun et al. (2019) demonstrated no statistically significance between the intervention group and control group [21], probably caused by the low number of participants. Our findings are consistent with the systematic review conducted by Kidd et al. (2019) reporting that physical performance in frail older adults was improved with interventions to increase physical activity [24]. Moreover, other randomized clinical trial studies (RCTs) demonstrated that exercise therapy was positively associated with improved function in frail older people [34, 35].

None of the studies examined the effects of interventions on psychological and social domains, and only one study evaluated the effects on the psychological domain, assessed with the HRQoL measure [22]. Frailty is a state that affects biological, psychological, and social domains, and which leads to increased vulnerability and adverse outcomes in psychological and social domains similar to those in the physical domain. Moreover, evidence shows that frailty is not only based on physical issues but is also related to psychological and social variables [36]. Thus, hospitalized frail elderly need a wide range of services that cover psychological and social components as well as interventions in the physical domain. Pérez et al., (2019) proposed a multidimensional intervention based on a group physical activity which improved social communication and psychological wellbeing among frail older adults in a primary care setting [33]. Finally, due to the multidimensional nature of frailty, a multidimensional care model for hospitalized frail older adults that covers physical, psychological and social components is needed [37].

The CGA intervention group led to a longer stay than the control group, but mHELP and APEP interventions did not show any statistical difference [21, 29, 30]. Most previous studies have reported associations between frailty and longer length of stay in hospital [38–41]. However, the cohort study by Engelhardt et al. (2018) demonstrated that frailty screening, followed by implementation of a frailty pathway, decreased the length of stay in hospital [42]. It seems that the reason for inconsistency in the studies is a difference in the aims of the interventions. The aim of frailty pathway intervention is to decrease of length of stay [42] whereas the aim of CGA intervention was improve physical functioning and to prevent frailty [29]. Thus, studies with different aims report diverse findings. The development of suitable interventions requires a comprehensive view of frailty and its consequences.

A decrease in re-hospitalization was demonstrated in the CGA unit intervention group [22]. Previous studies showed positive association between frailty and re-hospitalization [16, 20, 43]. It seems a thorough assessment of health problems was conducted during the hospitalization period which decreased re-hospitalization. This may cost-effective for health-service in long-term, however further studies on re-hospitalization and frailty are needed.

Only Ekerstad et al. (2017) evaluated the mortality rate and post discharge placement, showing that CGA was effective in reducing risk in these areas [22]. However, previous studies reported a high mortality rate and the increased use of additional services in frail elders [20, 44]. The mHELP and APEP studies did not address these outcomes. It appears that a classification of hospitalization outcomes is needed in clinical trial registration so that these can be evaluated in clinical trials.

Only Ekerstad et al. (2018) evaluated patient satisfaction with interventions [31]. Previous studies showed that patients with experience of improvement are satisfied with their care, while patients who experience poor health related quality of life are often dissatisfied [45, 46]. Satisfaction with an intervention is dependent on the effectiveness of the care process, and it can be an indicator of the suitability of an intervention [47]. Clinical trials would benefit from a satisfaction scale for the assessment of patients experiences of interventions.

Our study demonstrated that frailty was assessed by a variety of instruments: The FRESH screening tool [22], Frailty index [21], and Fried’s criteria [11, 30]. The FRESH screening tool was developed and validated by Eklund and colleagues. It was developed as a short screening instrument in acute care units [48] and focused on physical frailty [49]. Also, the Fried criteria, so-called the phenotype of frailty, focus on the physical domain whereby the psychological and the social domains of frailty are ignored [49]. The phenotype of frailty contains the following five criteria: unintended weight loss, weakness, low activity, and slowness. Using objective, physical measurements; this phenotype that had higher accuracy compared to self-report scales in relation to objective measurement of frailty [50]. The Frailty index, developed and validated by Rockwood and colleagues [51], is a more multidimensional measure of frailty, but it is time consuming to carry out [52]. Considering the disadvantages of these scales for hospitalized frail older adults, we need standard scales which are: multidimensional; quick to complete; nurse-led; not needing complex training; and which cover the contributing factors of frailty in hospital such as decline in sleep quality, delirium, disorientation, adaptation with hospital environment etc.

Screening of potentially eligible studies showed two methodological problems. Firstly, the use of nonspecific tools (such as Activities of daily living (ADL) and Instrumental activities of daily living (IADL)) to measure of frailty in clinical trial studies. Frailty is a new, complex and multidimensional concept, and unfortunately there is no gold standard for measurement [23]. This has led to use of various non-specific tools such as physical or functional tools that do not cover all aspects of frailty [23]. The second problem is that frailty is not considered an important outcome. In most of potentially eligible studies, frailty is evaluated as a predictor of other outcomes, and was only evaluated for comparison of two groups at baseline. Our findings are consistent with findings of previous studies [6, 53]. These methodological problems prevent the development of a comprehensive intervention protocol for caring of hospitalized frail older adults.

Interventions and new clinical trials in hospital care need to be revised according to the definition of frailty, and a new understanding of the needs of inpatient older adults. Many studies on interventions for frailty relate to medical and physiotherapy sciences rather than nursing care of hospitalized older adults. A reason of this may be low number of specialist geriatric nurses, poor training of nurses in this area, and a lack of clarity of multidimensional needs of hospitalized frail elders. The studies in this review point to the importance of specialist interventions, and the need for geriatric specialists in a multidisciplinary team such as geriatric nursing, geriatric physician, dietician, physiotherapist, occupational therapist etc.

Some limitations of our study should be mentioned. Only seven studies complied with the inclusion criteria. In addition, six of these studies showed poor quality due to high risk of bias. Future studies must pay more attention to international protocols such as Cochrane Risk of Bias Tool for clinical trial studies and CONSORT. It should also be noted that there was heterogeneity with regard to the included studies. For example, the operationalization of frailty differed between studies. Besides these limitations, the present study has many strengths. The main strength was a thorough and systematic approach (writing search strategies, comprehensive search, screening of studies, risk-of-bias appraisal and data extraction) by two independent researchers.

Due to the low number of RCTs on frailty conducted in a hospital setting, and low quality of existing studies, there is a need for further RCTs to examine a protocol appropriate for frail older people. This protocol should be developed based on qualitative studies that was covered multidimensional needs of frail elder peoples, elder friendly setting, interdisciplinary team, geriatric specialty of caring team and frailty evaluation with multidimensional hospital-based scale.

Interventions for hospitalized frail older adults are effective in the management of frailty. Multidimensional interventions conducted by a multidisciplinary specialist team on a geriatric ward is likely to be effective in the care of hospitalized frail elderly, because frailty is a condition that affects older people physically, psychologically, and socially.

## Supplementary Information

**Additional file 1.**

**Additional file 2.**

**Additional file 3.**

## Data Availability

We found seven studies on hospitalized frail older adults. The data can be found in Table 1. Also, these were published in peer-reviewed manuscripts, which are available on PubMED/MEDLINE.

## Abbreviations

ADL: Activities of daily living
ADLs: Activity daily living staircase
APEP: Augmented a prescribed exercise program
CGA: Comprehensive geriatric assessment
DEMMI: De Morton mobility index
ESDS: Enforce social dependency scale
EQ-VAS: EuroQol-visual analog scale
FAC: Functional ambulation categories
FES- І: Falls efficacy
GFK: Early rehabilitation in geriatric medicine
HABAM: Hierarchical assessment of balance
HRQoL: Health-related quality of life scale
HS: Handgrip strength
HUI-3: Health utilities index-3
IADL: Instrumental activities of daily living
MeSH: Medical subject headings
mHELP: Modified Hospital elder life program
Ni/Nc: Number of intervention group/number of control group
NNR: Needed to read
NU: NÄL-uddevalla
OR: Odds ratio
RCTs: Randomized clinical trial study
TUG: Timed up-and-go test
6-MWT: 6-Minute walk test
