# Supplementary material for: The impact of interventions on management of frailty in hospitalized frail older adults: a systematic review and meta-analysis
Source: BMC Geriatr. 2020 Dec 3;20:526. doi: 10.1186/s12877-020-01935-8 (PMC7712609; doi:10.1186/s12877-020-01935-8)
Supplement: Supplementary file 1 — Additional file 1. [file 12877_2020_1935_MOESM1_ESM.docx]

| Strategy search in PubMed | | |
| --- | --- | --- |
| Search | Queries | Number |
| 6 | ((((((((Elder*[tiab] AND Frail[tiab]) OR "Functionally-Impaired Elderly"[tiab] OR (Elderly[tiab] AND Functionally-Impaired[tiab]) OR "Functionally Impaired Elderly"[tiab] OR "Frail Elder"[tiab] OR Frail*[tiab] OR "Frailty syndrome"[tiab] OR (frail*[tiab] AND syndrome[tiab]) OR Prefrail*[tiab] OR Pre-frail*[tiab] OR "Frailty"[mh] OR "Frail elderly"[mh])))) AND ((Prevent*[tiab] OR Effec*[ti] OR Program*[ti] OR Outcome[ti] OR Intervention*[ti] OR efficacy[ti]))) AND (((Old*[tiab] OR elder*[tiab] OR aged[tiab] OR "aged"[mh] OR "aged, 80 and over"[mh]))) AND 2000:2019[dp]) NOT (((systematic review[tiab] OR meta analysis[tiab] OR meta-analysis[tiab] OR "narrative review"[tiab] OR review[ti] OR prevalence[ti] OR cross-sectional[ti]))) | [3684](https://www.ncbi.nlm.nih.gov/pubmed/?cmd=HistorySearch&querykey=45) |
| 5 | (systematic review[tiab] OR meta analysis[tiab] OR meta-analysis[tiab] OR "narrative review"[tiab] OR review[ti] OR prevalence[ti] OR cross-sectional[ti]) | [697759](https://www.ncbi.nlm.nih.gov/pubmed/?cmd=HistorySearch&querykey=44) |
| 4 | 2000:2019[dp] | [16423219](https://www.ncbi.nlm.nih.gov/pubmed/?cmd=HistorySearch&querykey=12) |
| 3 | (Old*[tiab] OR elder*[tiab] OR aged[tiab] OR "aged"[mh] OR "aged, 80 and over"[mh] | [4307799](https://www.ncbi.nlm.nih.gov/pubmed/?cmd=HistorySearch&querykey=5) |
| 2 | Prevent*[tiab] OR Effec*[ti] OR Program*[ti] OR Outcome[ti] OR Intervention*[ti] OR efficacy[ti] | [3610232](https://www.ncbi.nlm.nih.gov/pubmed/?cmd=HistorySearch&querykey=38) |
| 1 | ((Elder*[tiab] AND Frail[tiab]) OR "Functionally-Impaired Elderly"[tiab] OR (Elderly[tiab] AND Functionally-Impaired[tiab]) OR "Functionally Impaired Elderly"[tiab] OR "Frail Elder"[tiab] OR Frail*[tiab] OR "Frailty syndrome"[tiab] OR (frail*[tiab] AND syndrome[tiab]) OR Prefrail*[tiab] OR Pre-frail*[tiab] OR "Frailty"[mh] OR "Frail elderly"[mh]) | [23456](https://www.ncbi.nlm.nih.gov/pubmed/?cmd=HistorySearch&querykey=8) |
