# Supplementary material for: The impact of interventions on management of frailty in hospitalized frail older adults: a systematic review and meta-analysis
Source: BMC Geriatr. 2020 Dec 3;20:526. doi: 10.1186/s12877-020-01935-8 (PMC7712609; doi:10.1186/s12877-020-01935-8)
Supplement: Supplementary file 2 — Additional file 2. [file 12877_2020_1935_MOESM2_ESM.docx]

| **study** | **Random sequence generation** | **Allocation concealment** | **Selective reporting** | **Other sources of bias** | **Blinding of participants and personnel** | **Blinding of outcome assessment** | **Incomplete outcome data** | **Risk of Bias** |
| --- | --- | --- | --- | --- | --- | --- | --- | --- |
| (29) | High | Unclear | Unclear | High | Unclear | Unclear | Unclear | Poor |
| (32) | High | Unclear | Unclear | High | Unclear | Unclear | Unclear | Poor |
| (31) | High | Unclear | Unclear | High | Unclear | Unclear | Unclear | Poor |
| (22) | High | Unclear | Unclear | High | Unclear | Unclear | Unclear | Poor |
| (21) | Low | Low | Low | Low | High | Low | Low | Fair |
| (11) | Low | Low | High | Low | Unclear | Unclear | Low | Poor |
| (30) | Unclear | Unclear | Unclear | Low | High | Low | Low | Poor |
