# Supplementary material for: The impact of interventions on management of frailty in hospitalized frail older adults: a systematic review and meta-analysis
Source: BMC Geriatr. 2020 Dec 3;20:526. doi: 10.1186/s12877-020-01935-8 (PMC7712609; doi:10.1186/s12877-020-01935-8)
Supplement: Supplementary file 3 — Additional file 3. [file 12877_2020_1935_MOESM3_ESM.docx]

| **Duration of intervention** | **Experimental/control condition** | **study** |
| --- | --- | --- |
| Intervention period:  hospitalization time of participants  intervention group, (mean): 11.2  control group, (mean): 9.2 | **Experimental group:**  **Comprehensive geriatric assessment unit:**   - Department and facility:   Two acute elderly care (CGA unit); total of 48 beds; one, two or four-bed rooms; Division of Internal Medicine and emergency Care   - Team member:   Physician (specialists in internal medicine, family medicine and/or geriatrics}, licensed practicing nurses (Including specialized admission and discharge nurses), Occupational therapists, Physiotherapists, Counseling of nutritionists  A team conference is held every day, share information and experiences   - Treatment:   Systematic, structured interdisciplinary CGA and care by validated instruments focusing on the following: somatic and mental health, medication review, functional and activity ability including early rehabilitation, social situation, and early discharge planning  Person centered approach focusing with goal of keeping patient ambulatory and independent  Information is usually given to patient and their relatives  Advice regarding physical exercise after discharge   - Admission route:   Directly to the CGA unit ward via ambulance or primary care  **control group:**  **Caring in conventional acute medical care:**   - Department and facility:   Wards of internal and emergency medicine: one, two, or four bedrooms; Division of Internal Medicine and emergency Care   - Team member:   Physician (specialists in internal medicine), licensed practicing nurses, counseling of occupational therapists, counseling of physiotherapists, counseling of nutritionists   - Treatment:   Standard procedures according to national and international guidelines   - Admission route:   Via the emergency ward | (22, 29, 31, 32) |
| Intervention period: 12 weeks  Individual session: 4-5 sessions per week, each session 20 to 30 min | **Experimental group:**  **Usual care**   - Medical care and early rehabilitation according to GFK standards that were described in control group   **APEP (augmented prescribed exercise program)**   - Additional individual physiotherapy sessions that no pre-defined protocol or set of exercises - The starting level was defined according to the results of the individual CGA and the individual test scores - the selection, intensity and progression of the APEP physiotherapy sessions were directed by the exercise providers - Participants were encouraged to be active and to walk on the hospital ward as much as possible - Each session was scheduled for 20 to 30 min and followed the same structure:  1. Brief assessment of the participant’s recent condition, motivation and recovery level 2. Warm up (3 min): low-intensity chair- based exercises to stimulate the circulation or by walking to the ward therapy room. 3. Main part (15–20 min): t least three strengthening exercises, followed by 2–3 balance, endurance and/or walking exercises 4. Cool-down (2 min): walking back to the participant’s ward room 5. Brief post-test, including BORG assessment of session perceived exertion   **control group:**   - Medical care - Early rehabilitation according to GFK, GFK is:   Multidisciplinary team: including medical doctors, nurses, physiotherapists, occupational therapists, speech and language therapists and other allied health professionals  A geriatric assessment immediately after admission and at discharge  Assessment of the patient’s social and environmental situation  Multidisciplinary team meetings once a week  Activating-therapeutic nursing care  At least two of the following four treatments: physiotherapy/physical therapy, occupational therapy, speech and language therapy, psychology/neuro-psychology | (21) |
| Intervention period: hospitalization period of participants  mHELP sessions (11):   - 7 days in period, 1.7 sessions per day and 30 min per sessions   mHELP sessions (30):   - Mean 7 days intervention - 30 to 45 min per da | **Experimental group:**  **Usual care**   - were described in control group   **mHelp program**  early mobilization:   - Assess patient ability - Physical assist patient carrying out activities (from range of motion to ambulation) - Frequency (Duration): 3 times a day (varies based on patient tolerance)   oral and nutritional assistance:   - daily oral care; tooth brushing and oral-facial range-of-motion exercises for lips/tongue/jaw - diet education for postsurgical intake - Frequency (Duration):daily oral care, surveillance at breakfast and lunch time (based on patient needs)   orientation communication:   - active orientation - Frequency (Duration): once daily (around 20 min, mostly incorporate into mobilization)   **Control group:**  **Preoperative**   - standard hospital care provided by physicians and nurses - all participants received the study site’s routine standardized - mechanical bowel preparation - nothing-by-mouth (NPO) for at least 8 hours   **Postoperative**   - oral intake was withheld until the return of first flatus or defecation - first nutrition a clear liquid diet that progressing to a regular diet as tolerated - encouraged to ambulate and did so as tolerated - Additional care from a dietician or physical therapist was only provided at the request of the attending physicians | (11, 30) |
| CGA: Comprehensive Geriatric Assessment, GFK: early rehabilitation in geriatric medicine, APEP: augmented prescribed exercise program | | |
